# Dielectric, Ferroelectric, and Magnetic Properties of Sm-Doped BiFeO_3_ Ceramics Prepared by a Modified Solid-State-Reaction Method

**DOI:** 10.3390/ma11112208

**Published:** 2018-11-07

**Authors:** Fuzeng Zhang, Xiangjun Zeng, Daoguang Bi, Kailong Guo, Yingbang Yao, Shengguo Lu

**Affiliations:** 1 Electric Power Research Institute, China Southern Power Grid Co., Ltd., Guangzhou 510080, China; zhangfz@csg.cn (F.Z.); zengxj@csg.cn (X.Z.); 2 School of Materials and Energy, Guangdong University of Technology, Guangzhou 510006, China; m13113987120@163.com (D.B.); 15521029043@163.com (K.G.); sglu@gdut.edu.cn (S.L.)

**Keywords:** BFO, Sm dopant, dielectric, ferroelectric, magnetic

## Abstract

Sm-doped BiFeO_3_ (BFO) material was prepared using a modified solid-state-reaction method, which used fast heating and cooling during the sintering process. The Sm doping level varied between 1 mol % to 8 mol %. Processing parameters, such as sintering temperature and annealing temperature, were optimized to obtain high-quality samples. Based on their dielectric properties, the optimum sintering and annealing temperatures were found to be 300 °C and 825 °C, respectively. Leakage-free square-shaped ferroelectric hysteresis loops were observed in all samples. The remnant polarization was maximized in the 5 mol %-doped sample (~35 μC/cm^2)^. Furthermore, remnant magnetization was increased after the Sm doping and the 8 mol%-doped sample possessed the largest remnant magnetization of 0.007 emu/g. Our results demonstrated how the modified solid-state-reaction method proved to be an effective method for preparing high-quality BiFeO_3_ ceramics, as well as how the Sm dopant can efficiently improve ferroelectric and magnetic properties.

## 1. Introduction

In the past decade, considerable attention has been devoted to the study of multiferroics BiFeO_3_ (BFO) material, which in the same phase exhibit a simultaneous presence of ferroelectricity and (anti)ferromagnetism. The material shows great potential in applications of information storage, spintronics, and sensors [1]. Moreover, it has a high ferroelectric Curie temperature of ~830 °C and antiferromagnetic Néel temperature of ~370 °C [2]. BFO has an antiferromagnetic spin ordering and shows no remnant magnetization [1,2,3]. The ferroelectric property of BFO single crystals was shown to have a saturated polarization of 100 μC/cm^2^ along the [001]_hex_ direction [4]. However, for bulk ceramics, the leakage problem, caused by defects and nonstoichiometry, has limited the applications of this material. Because of the volatility of bismuth and the instability of iron valence state, it is hard to obtain ideal pure-phase BFO [4,5]. Recently, the liquid phase sintering method has been shown to be an effective way to obtain leakage-free and pure phase BFO materials [6]. In this method, a heating rate as high as 100 °C/s and a short sintering time of 5 min were applied to obtain high-insulating and pure phase BFO ceramics. However, this is only feasible for small-dimensioned samples, since for larger samples such short time is not sufficient for thermal equilibrium. Moreover, such high heating rate is out of the capability of a conventional furnace. 

Walker et al. used conventional solid-state and mechano-chemical activation-assisted solid-state synthesis methods to prepare Sm-doped BFO ceramics (Bi_0.88_Sm_0.12_FeO_3_) and found that the synthesis methods considerably affects the crystal structures as well as the ferroelectric properties [7]. Verma et al. prepared Sm-doped BFO ceramics (Bi_0.9_Sm_0.1_FeO_3_) using a sol-gel routine and found that the sample was too leaky to obtain reasonable ferroelectric hysteresis loop [8]. Tao et al. obtained pure-phase Sm-doped BFO ceramics (Bi_1-x_Sm_x_FeO_3_, x = 0.05, 0.075, 0.1, 0.125, 0.20, 0.25) using a conventional solid-state method and found that the rhombohedral phase favors (x = 0.05, 0.075) the piezoelectric properties [9]. Therefore, a more efficient method should be explored with an emphasis on cost and feasibility. Herein, we report a simple method based on the conventional solid-state-reaction method that prepares high-quality BFO ceramics.

Another issue concerning the application of BFO material is the zero remnant magnetization (Mr) as a result of the antiferromagnetic spin ordering. This is because for the magnetoferroelectric (ME) applications, the Mr is a must for the ME coupling to occur [3,10,11,12,13,14,15]. The usual way to produce Mr in BFO material is by doping another cation at the Bi^3+^ site, either magnetically-active ions (Nd, Sm, Gd, Tb, Mn, Ni, Co) or non-magnetically-active ions (La, Ca, Sr, Ba, Pb, Pd, Ti, Al, Zn) [14,15,16,17,18,19,20,21,22]. The remnant magnetization can also be induced by doping at the Fe^3+^ site. Meanwhile, the antiferromagnetic Néel temperature will be decreased by the doping [23,24]. Herein, we optimized a modified solid-state-reaction method and obtained high-quality Sm-doped BFO ceramics. 

It was previously found that the La-doped BFO undergoes structural transformation from rhombohedral to triclinic or orthorhombic around 10 mol % [9,11]. Sm^3+^ possesses smaller ionic radius compared to La^3+^. Kan et al. found that a smaller ionic radius at the Bi-site of the rare-earth doped BFO will induce antiferroelectric-like double-hysteresis P–E loops [25]. Moreover, at high doping levels, rare-earth elements with a smaller ion radius favors the formation of paraelectric orthorhombic phase [25]. Tao et al. reported the doping levels, at which the rhombohedral-orthorhombic phase boundary was formed for La (10 mol %), Pr (10 mol %), Eu (10 mol %), and Sm (7.5 mol %) [9]. Karimi et al. found that when La, Nd, Sm, and Gd-doped BFO ceramics had doping levels below 10 mol %, all the doped samples exhibited rhombohedral phase and with increasing the doping level, for Nd and Sm cases, an intermediate phase (antiferroelectric) was formed [26]. This was related to the average ionic radius at the Bi-site [26]. 

Arnold et al. reviewed the structural phase transitions in rare-earth doped BFO materials and found that average Bi-site ionic radii are the primary driving forces for phase transitions, and that synthesis methods considerably account for the controversy in the literature [27]. Yang et al. suggested that the formation and stability of the intermediate phase is related to a flexoelectric interaction induced by the rare-earth dopants, which stabilizes complex modulated phases through domain wall energy minimizations [3]. Recently, Walker et al. reported a large strain (0.3%) in Sm-doped BFO ceramics near the morphotropic phase boundary (MPB) at the doping level of ~15 mol % [28]. Electric-field induced phase transition of the intermediate phase was suggested to play an important role in such high strain response [28]. Yu et al. used spark plasma sintering method to prepare Bi_1−x_Sm_x_FeO_3_ (x = 0.15–0.18) ceramics and verified the existence of three different phases: ferroelectric *R3c*, antiferroelectric *Pnam*, and paraelectric *Pnma* [29]. However, their samples were very leaky and the P–E loops were round-like [29]. Based on the survey in literature, we found that for Sm dopant, when the doping level is below 10 mol %, the phase is pure ferroelectric *R3c* phase. Additionally, in this doping range the electrical properties of the doped BFO ceramics, for example the piezoelectric coefficients, were better than those heavily doped [9]. The compositions need to be finely tuned further and deserve more detailed study.

Therefore, in current study the doping level was set to be 1 mol %, 3 mol %, 5 mol %, and 8 mol % (denoted as Sm1, Sm3, Sm5, and Sm8 in the context) in order to rule out the lattice structure effects. There were two effects expected for the Sm dopants: first, that it would substitute the bismuth ions; second, that it may partially destroy the antiferromagnetic cycloidal spiral structure which may result in produce remnant magnetization [4,30]. Our results show that Sm dopant can improve the dielectric, ferroelectric and magnetic properties of BFO ceramics.

## 2. Materials and Methods

**Figure 1:**
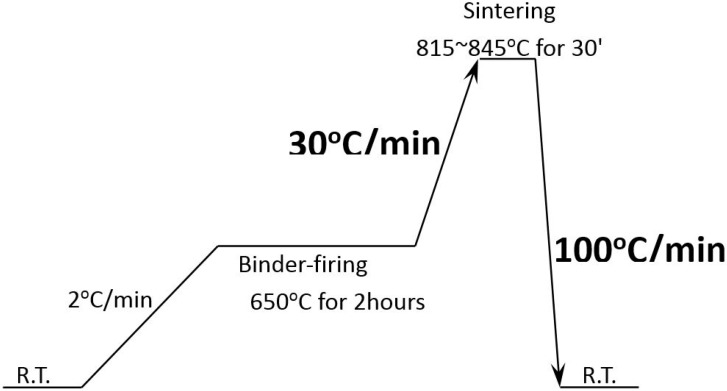
Schematic illustration of the sintering process.

Stoichiometric amounts of high purity Bi_2_O_3_ and Fe_2_O_3_ (purity: 99.9%) were weighed and ball-milled (QMJ-A, NBD Materials Science and Technology Co., Zhengzhou, China) in ethanol alcohol for 24 h. The mixture was calcined (NBD-M1700, NBD Materials Science and Technology Co., Zhengzhou, China) at 650 °C for 5 h in air followed by furnace cooling. The calcined powders were ground and ball-milled again for 48 h together with the additive of Sm_2_O_3_ (purity: 99.99%). The amount of Sm_2_O_3_ was 1 mol %, 3 mol %, 5 mol % and 8 mol % relative to the BFO raw powders. Then, polyvinyl alcohol (PVA) was added into the dried powders as a binder. The powders were pressed (uniaxially, 200 MPa, YLJ-15T, Hefei Ke Jing Materials Technology Co., Hefei, China) into disks with a diameter of 10mm. After burning the binder at 650 °C for 2 h, the samples were sintered. The sintering temperature varied between 815–845 °C, with an interval of 5 °C. During the sintering process, we adopted a much higher heating/cooling rate (heating: 30 °C/min, cooling: 100 °C/min) than the conventional method (~2 °C/min), as shown in Figure 1. The heating rate was reached using the conventional furnace. In order to get to the high cooling rate, the furnace was opened as soon as the sintering finished. After sintering, the samples were annealed in air at different temperatures ranging from 100–500 °C for 5 h. The optimum sintering temperature and the annealing temperature were obtained after analyzing the properties of these samples prepared under different conditions.

X-ray diffractometer (X’pert System, Philips, Almelo, The Netherland) with a Cu Kα source and field-emission scanning electron microscope (JEOL JSM-6335F, Tokyo, Japan) were used to study the crystal structures and microstructures, respectively. The Raman scattering spectroscopy (Horiba Jobin Yvon, HR800, Paris, France) were taken at room temperature with backscattering geometry. For electrical measurements, the samples were thinned to 0.3~0.4 mm plates and silver electrodes (2 mm in diameter) were fired on both sides The dielectric properties were measured on an HP4294A impedance analyzer (Hewlett-Packard, CA, USA). The ferroelectric hysteresis loops were taken on a modified Sawyer-Tower circuit (Homemade, Guangzhou, China) and the measurement frequency was set to 100 Hz. A sinusoidal waveform (Keysight 33210A, Keysight, Shanghai, China) was used for the applied electric field. No pre-poling was applied. A vibrating sample magnetometer (Lakeshore 7400 series, Lake Shore Cryotronics, Inc., Westerville, OH, USA) was used to characterize the magnetic properties.

## 3. Results and Discussion

### 3.1. Optimization of Sintering and Annealing Temperatures

**Figure 2:**
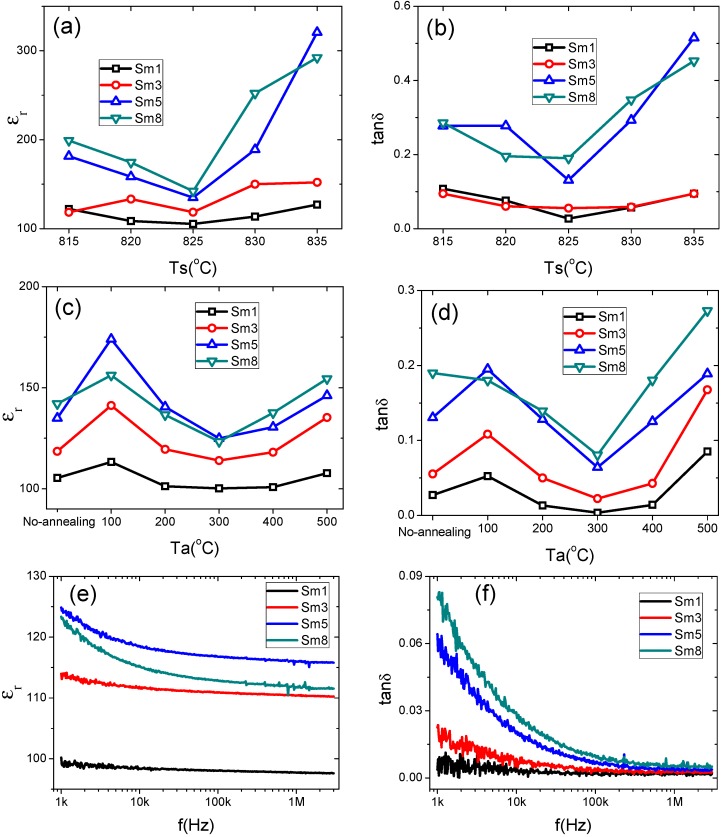
Sintering temperature (T_s_) effects on the (**a**) relative dielectric constant ε_r_ and (**b**) dielectric loss tan δ; annealing temperature (T_a_) effects on the (**c**) ε_r_ and (**d**) tan δ of Sm-doped BFO ceramics. The results were measured at 1 kHz. (**e**) and (**f**) show the frequency (**f**) dependency of ε_r_ and tan δ of the Sm-doped samples after sintering at 825 °C and annealing at 300 °C.

Figure 2a,b shows the dielectric constants and dielectric losses (at 1 kHz), respectively, of the samples sintered at different temperatures. Higher sintering temperatures over 835 °C introduced lots of cracks and pores to the samples and the leakage was too large to effectively measure the dielectric response. The results for these cracked and porous samples are not shown here. As indicated in Figure 2a, in all samples the dielectric constant was initially decreased with sintering temperatures ranging from 815–825 °C, and then increased with sintering temperatures ranging from 825–835 °C. The same trend was observed in the dielectric loss, as shown in Figure 2b. Such behavior is independent of the Sm doping levels. For example, in Sm5 sample, the dielectric constant/loss (1 kHz) was 181/0.28 when sintered at 815 °C, then decreased to 135/0.13 when sintered at 825 °C, and maximized to 321/0.51 as the sintering temperature increased to 835 °C. Considering that the melting point of Bi_2_O_3_ was around 830 °C [5], we attributed the lower dielectric constant/loss of samples sintered at 825 °C to the effects of liquid phase Bi_2_O_3_. 

The liquid phase bismuth oxide facilitated the BFO phase formation and produced a dense microstructure. However, increasing the sintering temperature led to the bismuth loss and bismuth vacancies, as well as oxygen vacancies [4,5,6]. Such vacancy defects will increase the dielectric loss. It has been shown that the mobility of defect such as oxygen vacancies will greatly affect the domain wall pinning and thus result in different ferroelectric and dielectric performances [31]. Besides the effects from oxygen vacancies, the secondary phase also considerably affected the electrical properties of BFO ceramics [31,32]. Temperature-dependent XRD studies have shown that BFO is metastable, with respect to Bi_2_Fe_4_O_9_ and Bi_25_FeO_39_ [32]. Furthermore, these secondary phases grow with sintering temperatures above 600 °C and then react back to BFO at higher temperatures (above 850 °C) [33]. In current case, both of these defects and secondary phase formation affected the dielectric properties of our samples, and more investigations are needed to reveal the detailed mechanisms. In addition, the dielectric constant/loss increased by the Sm-dopant, especially as the doping level is over 3%, as shown in Figure 2a,b. The dielectric constant was increased from 105 to 142 and the loss was increased from 0.03 to 0.19 for the Sm1 and Sm8 samples sintered at 815 °C, respectively. From these results, the optimum sintering temperature was found to be 825 °C.

In order to further improve the dielectric property, the 825 °C-sintered samples were annealed in air at different temperatures ranging from 10–500 °C with an interval of 100 °C. The dielectric constant and dielectric loss after annealing are shown in Figure 2c,d, respectively. The dielectric constant/loss was minimized after annealing at 300 °C regardless of the Sm doping levels, as shown in Figure 2d. For example, the dielectric constant/loss (1 kHz) in Sm5 sample was reduced from 135/0.13 to 125/0.06 after annealing at 300 °C. The annealing can reduce oxygen vacancies. Therefore, the dielectric loss is reduced. However, higher annealing temperatures may induce the decomposition of second phase in the ceramics, producing more oxygen vacancies [6], and eventually increase the dielectric loss. Another possible mechanism is the redistribution of oxygen vacancies after annealing at 300 °C, which might cause domain wall pinning and thus suppress the dielectric loss [31].

Based upon the dielectric measurements results, the optimum processing conditions can be summarized as below: sintering at 825 °C for 30 min followed by annealing at 300 °C for 5 h. The calcination temperature did not show prominent effects on the phase formation as well as properties of BFO ceramics.

The frequency dependence of dielectric constants and losses of samples prepared under the optimized conditions are shown in Figure 2e,f, respectively. The dielectric constant/loss was almost independent of frequency in Sm1 and Sm3 samples. The Sm1 exhibited the lowest dielectric loss ~0.01 in the whole frequency range from 1 kHz to 3 MHz. The frequency dispersion was increased significantly as the doping level larger than 3%, as evidenced in the Sm5 and Sm8 samples. The frequency dispersion of dielectric behavior at this frequency range was determined by the dipoles [33]. It will be shown later that the Sm5 and Sm8 samples possess larger remnant polarizations.

The dielectric properties of the samples prepared by this method were better than those of the Sm-doped BFO ceramics prepared by normal solid-state method or sol-gel method (larger dielectric constant, lower dielectric loss, lower frequency-dispersion), and comparable to those of the samples prepared by spark plasma sintering method. The dielectric constant and dielectric loss, at 1kHz, for the sol-gel derived Sm-doped BFO ceramics (Bi_0.9_Sm_0.1_FeO_3_) were around 125 and 0.40, respectively [8]. Furthermore, for the samples prepared from conventional solid-state method (Bi_1−x_Sm_x_FeO_3_, x = 0.05, 0.075), the dielectric constant and dielectric loss are around 60 and 0.01, respectively [9]. For the samples prepared by spark plasma sintering method (Bi_0.85_Sm_0.15_FeO_3_), the dielectric constant and dielectric loss are 150 and 0.01, respectively [29].

### 3.2. Microstructure and Phase Purity

**Figure 3:**
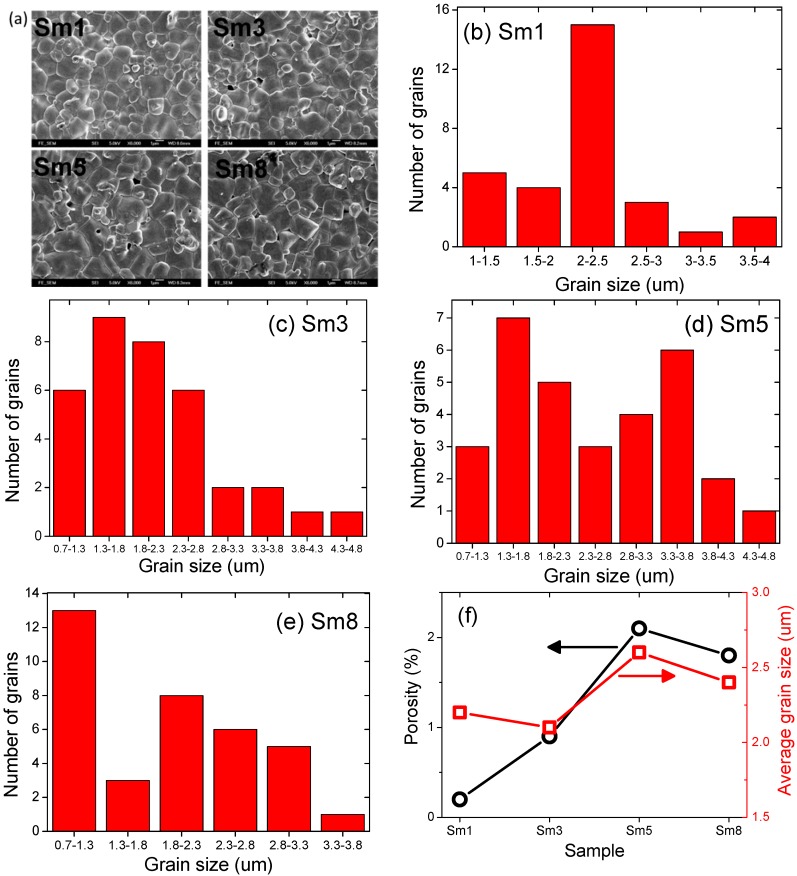
(**a**) SEM plane-views, (**b**–**e**) grain size distribution of Sm1–Sm8 samples, and (**f**) average grain size and porosity.

**Figure 4:**
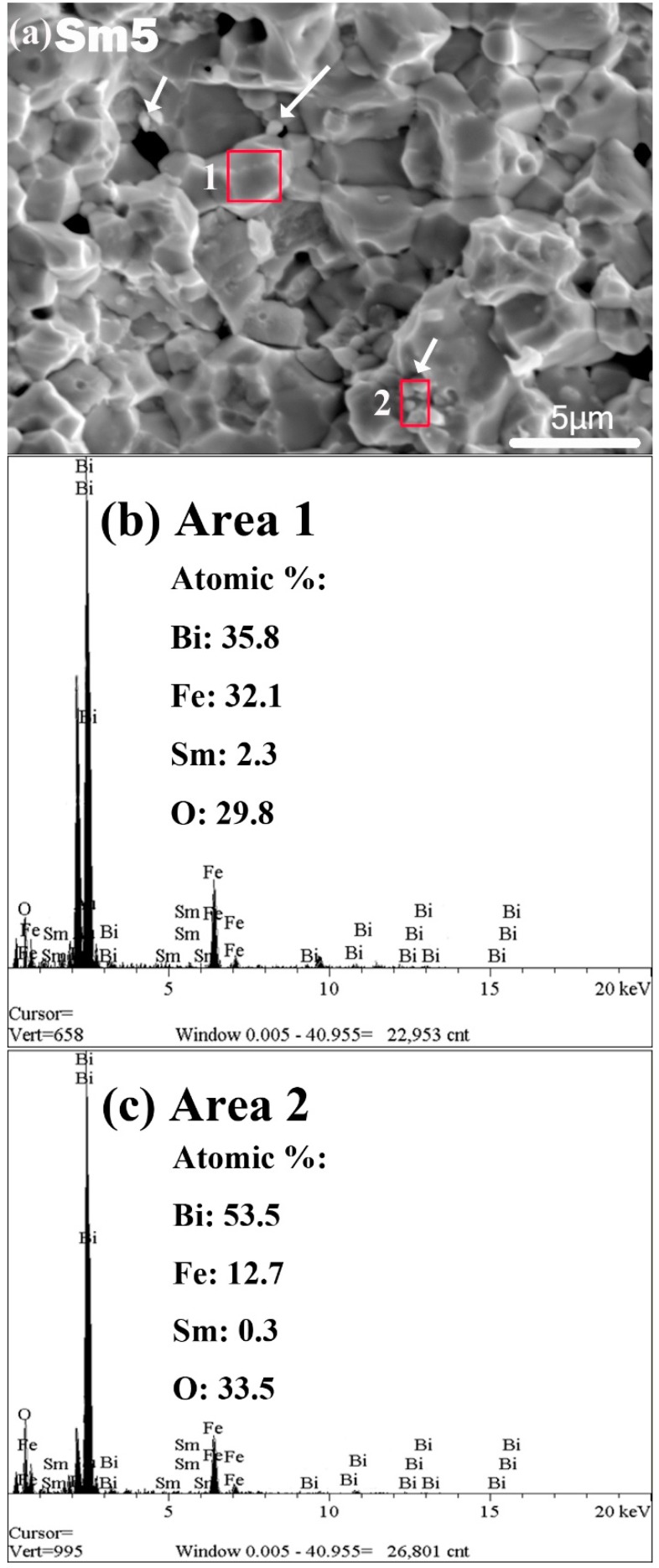
(**a**) SEM cross-section view of Sm5 sample, (**b**,**c**) are the EDX spectra obtained from the area 1 and area 2 as indicated in (**a**). The arrows in (**a**) indicates the Bi-rich secondary phase.

The SEM plane-views of the 825 °C-sintered samples are shown in Figure 3a. The grain size distributions are shown in Figure 3b–e for the Sm1, Sm3, Sm5, and Sm8 sample, respectively. The average grain size and percentage of porosity are shown in Figure 3f. The grain size is more uniform in the Sm1 sample, and with increasing Sm doping level, the grain size distribution became more scattered, as shown in Figure 3b–e. The average grain size was 2.2, 2.1, 2.6, and 2.4μm for the Sm1, Sm3, Sm5, and Sm8 sample, respectively. Additionally, the percentage of porosity was 0.2%, 0.9%, 2.1%, and 1.8% for the Sm1, Sm3, Sm5, and Sm8 sample, respectively, as shown in Figure 3f. Generally speaking, a high Sm doping level leads to larger grain size and more pores. This can account for the larger dielectric loss previously observed in higher Sm dopant samples. In order to check the formation of a secondary phase, we did the energy dispersive spectroscopy (EDX) on the fracture surfaces of all samples. Figure 4 shows the results for Sm5 sample. The EDX results show that the Bi-rich secondary phase was identified as small irregular-shaped particles located at the grain boundaries of the main phase.

**Figure 5:**
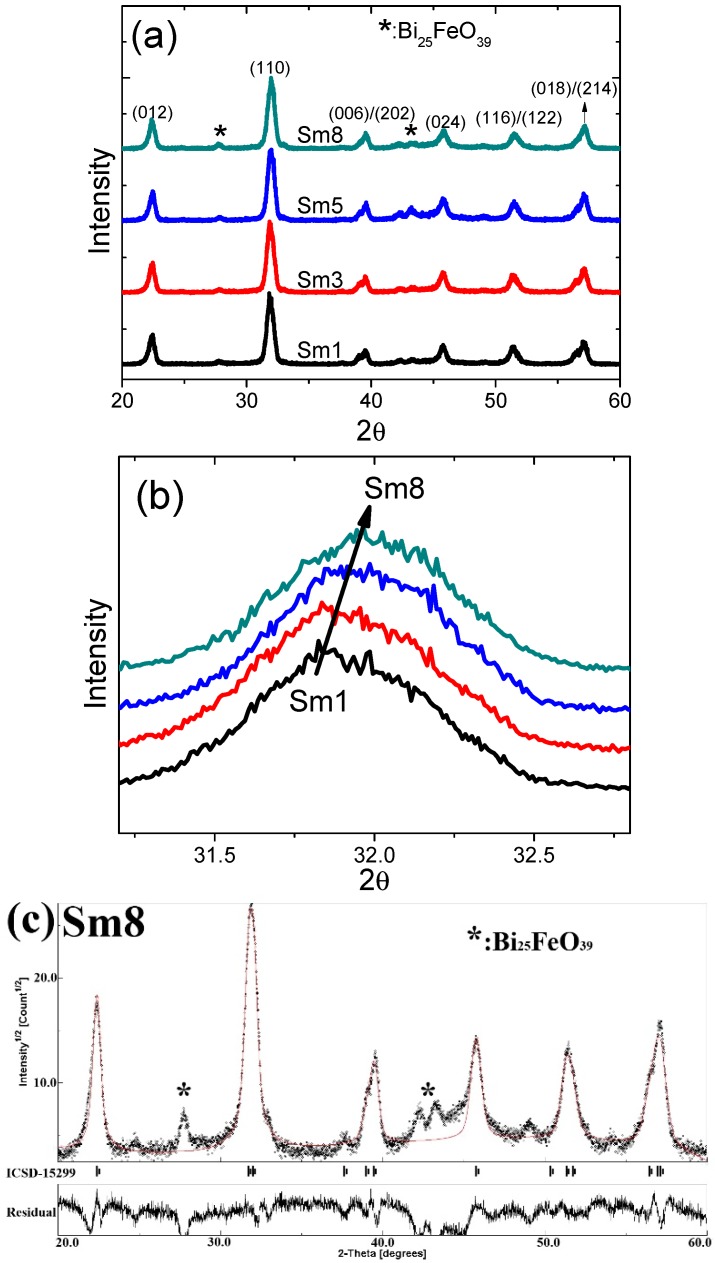

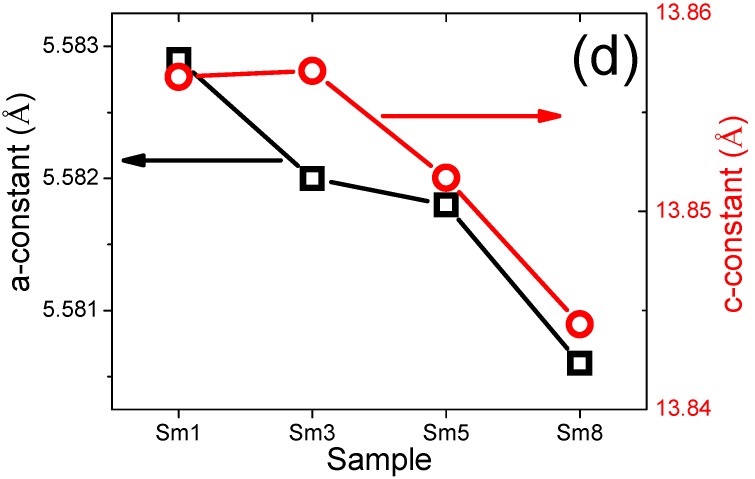
(**a**) XRD diffraction patterns, (**b**) enlarged view of the (110) peaks, (**c**) Rietveld refinement results of the Sm8 sample, (**d**) calculated lattice constants (a and c) of the Sm-doped BFO samples

The XRD θ–2θ diffraction patterns of these samples were shown in Figure 5a. All the diffraction peaks can be indexed as a rhombohedral structure [14] and the only second phase ~Bi_25_FeO_39_ (indicated by arrows in the figure) was observed. No traces of Sm_2_O_3_, Fe_2_O_3_, or SmFeO_3_ were observed. This suggests that Sm^3+^ has been dissolved into the lattices of BFO to form a normal rhombohedral structure. The diffraction peaks shift to higher 2θ values as the Sm dopant level was increased. For example, the major peak (110) shifts from 31.84° for Sm1 sample to 31.95° for Sm8 sample, as shown in Figure 5b. This implies that the lattice constant is decreased by the Sm dopant since the radius of Sm^3+^ (0.958 Å) is smaller than that of Bi^3+^ (1.030 Å). In order to further confirm this, we did the Rietveld refinement using the MAUD software [34] and ICSD#15299 (Rhombohedral *R3c* space group) was used for the fitting [7,9]. The refinement results are shown in Figure 5c for the Sm8 sample with sig = 1.89 and R_WP_(%) = 25.93. The large values of Rwp comes from the signals of the secondary phase (Bi_25_FeO_39_), as indicated in Figure 5c. Besides the secondary phase, the fitting was matches well with the experimental data, confirming the rhombohedral phase (*R3c*). The refinement results for other Sm-doped samples exhibit very similar behavior. The calculated lattice constant-a/c were obtained from the Rietveld refinement, which are 5.5829 Å/13.8568 Å, 5.5820 Å/13.8571 Å, 5.5818 Å/13.8517 Å, and 5.5806 Å/13.8443 Å for the Sm1, Sm3, Sm5, and Sm8 samples, respectively, as plotted in Figure 5d. The calculated unit cell volume were 388.4 Å^3^, 387.5 Å^3^, 387.9 Å^3^, and 385.5 Å^3^ for the Sm1, Sm3, Sm5, and Sm8 sample, respectively. The refinement results confirmed that the Sm dopant leads to the decrease of the lattice constants (a and c) as well as the unit cell volume.

**Figure 6:**
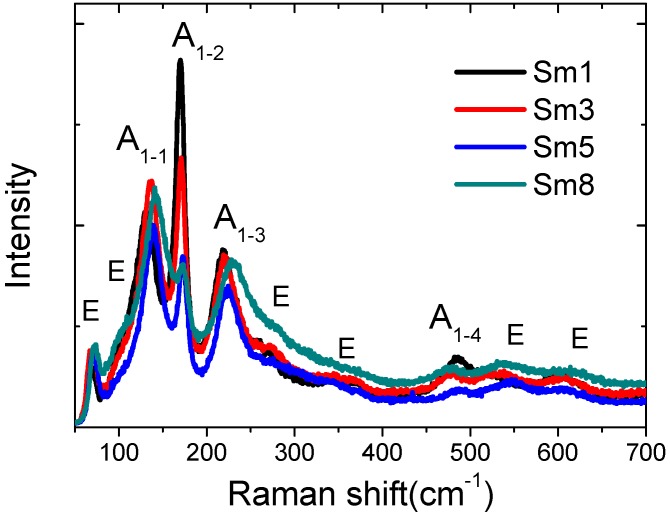
Raman spectra of Sm-doped BFO samples.

The Raman scattering spectra are shown in Figure 6. All the peaks are consistent with the published results [35]. No visible peaks from second phase were observed. Since Raman scattering spectra are sensitive to atomic displacements, the evolvement of Raman normal modes with the material compositions can provide valuable information about ionic substitution and electric polarization. These normal modes are dominated by the Bi-O bonds, especially in the three strongest A1 modes at 130 cm^−1^, 170 cm^−1^, and 219 cm^−1^, denoted as A1-1, A1-2, and A1-3 in Figure 6, respectively [35]. These peaks shift to higher frequencies with Sm dopant: from 130.5 cm^−1^/170.1 cm^−1^/218.6 cm^−1^ to 141.4 cm^−1^/173.2 cm^−1^/229.9 cm^−1^ for Sm1 and Sm8 samples, respectively. This frequency shift confirms that the Sm substitutes for Bi in the BFO samples: since the Sm element (atomic weight: 150.36) is lighter than Bi (atomic weight: 208.98), Sm doping will decrease the effective mass and increase the frequency of the normal modes ($f\propto \sqrt{m/k}$, where *m*: mass, *k*: force constant) [35].

### 3.3. Ferroelectric and Magnetic Properties

**Figure 7:**
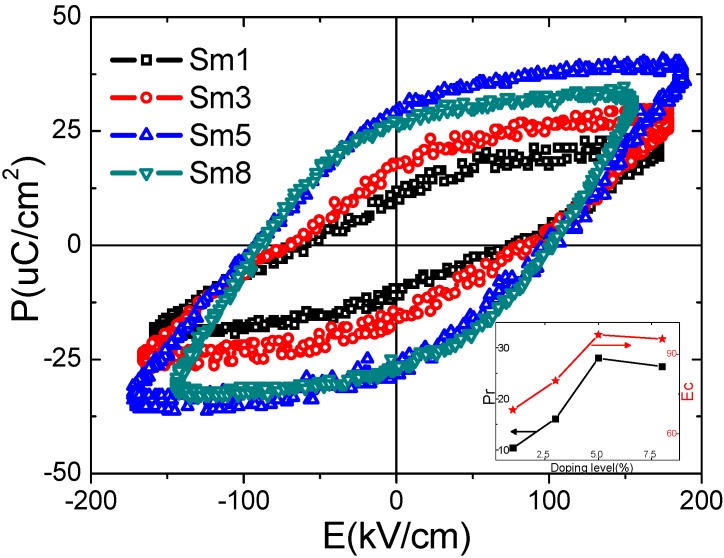
The ferroelectric P–E hysteresis loops of the 825 °C-sintered samples. The insets in the figure show the composition dependency of remnant polarization/coercive field (P_r_/E_c_).

The ferroelectric polarization vs. electric field (P–E) hysteresis loops of the 825 °C-sintered samples are shown in Figure 7. The measurement frequency was 100 Hz. Obviously, these hysteresis loops were not saturated due to the leakage problems at high electrical field. The remnant polarization (P_r_) was increased from 10.5 μC/cm^2^ for Sm1 sample to 16.1 μC/cm^2^ for Sm3 sample, and maximized at 28.0 μC/cm^2^ in the Sm5 sample, then slightly decreased to 26.3 μC/cm^2^ as the doping level further increased to 8 mol %. The coercive fields (E_c_) also exhibit such trend, changing from 69.0 kV/cm, 80.0 kV/cm, 97.3 kV/cm to 95.6 kV/cm for Sm1, Sm3, Sm5, and Sm8, respectively. The inset in Figure 7 summarized the results of the P_r_ and E_c_. Moreover, the P_r_ can be further increased up to 35.0 μC/cm^2^ (E_c_ = 98.0 kV/cm) in the Sm5 sample as the sintering temperature increased to 835 °C. 

**Figure 8:**
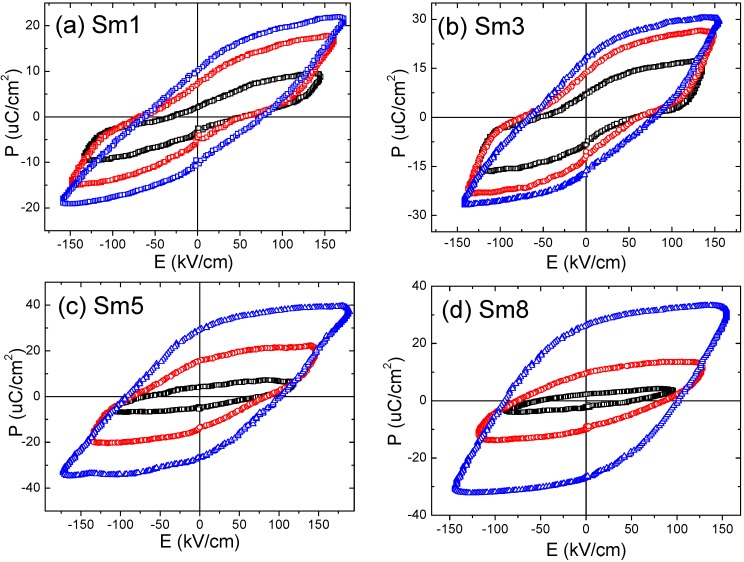
The ferroelectric P–E hysteresis loops of the (**a**) Sm1, (**b**) Sm3, (**c**) Sm5, and (**d**) Sm8 samples under different electrical fields. These samples were all sintered at 825 °C and post-annealed at 300 °C.

In the Sm1–Sm5 samples, the hysteresis loops under lower electric field (<120 kV/cm) showed resemblance of antiferroelectric (AFE) double-hysteresis loops (as shown in Figure 8), which has been observed by other researchers studying BFO materials [3,4,6,25,26,27,28,29,31]. Such behavior is more prominent in the Sm1 and Sm3 samples, as shown in Figure 8a,b, and diminishes gradually with increasing the Sm doping levels, as shown in Figure 8c,d. 

Under higher electrical field, the hysteresis loops became normal-ferroelectric like, as shown in Figure 8. For the Sm8 sample, such behavior was not observed, regardless if the applied field was low or high. Usually there are two kinds of mechanisms that account for the double hysteresis loops observed in BFO materials: one is the appearance of the intermediate antiferroelectric *Pnam* phase determined by the rare-earth doping levels [3,25,26,27,28,29], and the other is the defects arrangement or domain wall pinning effects [31,36]. In our case, due to the low doping level, all the samples exhibit rhombohedral phase only and no antiferroelectric intermediate phase was formed, as discussed above in the XRD section. Therefore, the origin of these double-hysteresis loops was believed to come from the effects of defects which restore the ferroelectric domain as the external electric field goes to zero [31,36]. 

In the current case, the defects dipoles should be related with the bismuth and oxygen vacancies. The positively charged oxygen vacancy ($V_{O}^{••}$) and the negatively charged bismuth vacancy ($V_{Bi}^{\prime \prime \prime }$) form defect dipoles, which provides the restoring force to reverse the polarization upon removal of external electric field. However, under the high electrical field, the defect dipoles were forced to align with the polarization, thus indicating that the restoring force disappears, as does the double hysteresis. 

Rojac et al. showed that quenching from temperatures higher than the Curie temperature or Bi-loss caused by high-temperature annealing suppresses the double-hysteresis behavior in undoped BFO ceramics [31]. The reason is that the defects were frozen when fast cooling from high temperatures, and frozen defects will not lead to domain-wall pinning. Thus, the double-hysteresis behaviors can be eliminated [31]. Such quenching effects were also observed in the Cu-doped K_0.5_Na_0.5_NbO_3_ ceramics by Lin et al [36]. Lin et al. also found that at high measurement temperatures or low measurement frequencies such double-hysteresis will diminish [36].

In our case, the double-hysteresis behavior is field-dependent—i.e., a high electric field will eliminate such behavior, and moreover, the Sm dopant can suppress such behavior. Our samples were prepared by fast cooling from a high temperature of 825 °C and then followed by post-annealing at a medium temperature of 300 °C. Therefore, the first step (quenching from high-temperature) will lead to frozen defects and suppress the double-hysteresis behavior. Meanwhile, the second step (annealing at a lower temperature) will lead to the migration/redistribution of the defects and cause a domain-wall pinning, favoring the double-hysteresis behavior. Therefore, these two competing mechanisms will account for the observed hysteresis shown in Figure 8. 

Based upon the above discussions, it can state that the Sm dopants may suppress the migration/redistribution of the defects, and hence the absence of the double hysteresis loops in the Sm8 sample. This hypothesis can be partially supported by the dielectric constants/losses shown in Figure 2e,f. By increasing the Sm doping level, the dielectric constants and losses were shown to increase; such behavior was very similar to the undoped BFO ceramics after quenching from high temperature where the defects were frozen (no redistribution/migration of defects) and domain wall pinning was suppressed [31]. More deep investigations are needed in order to elucidate the origins.

**Figure 9:**
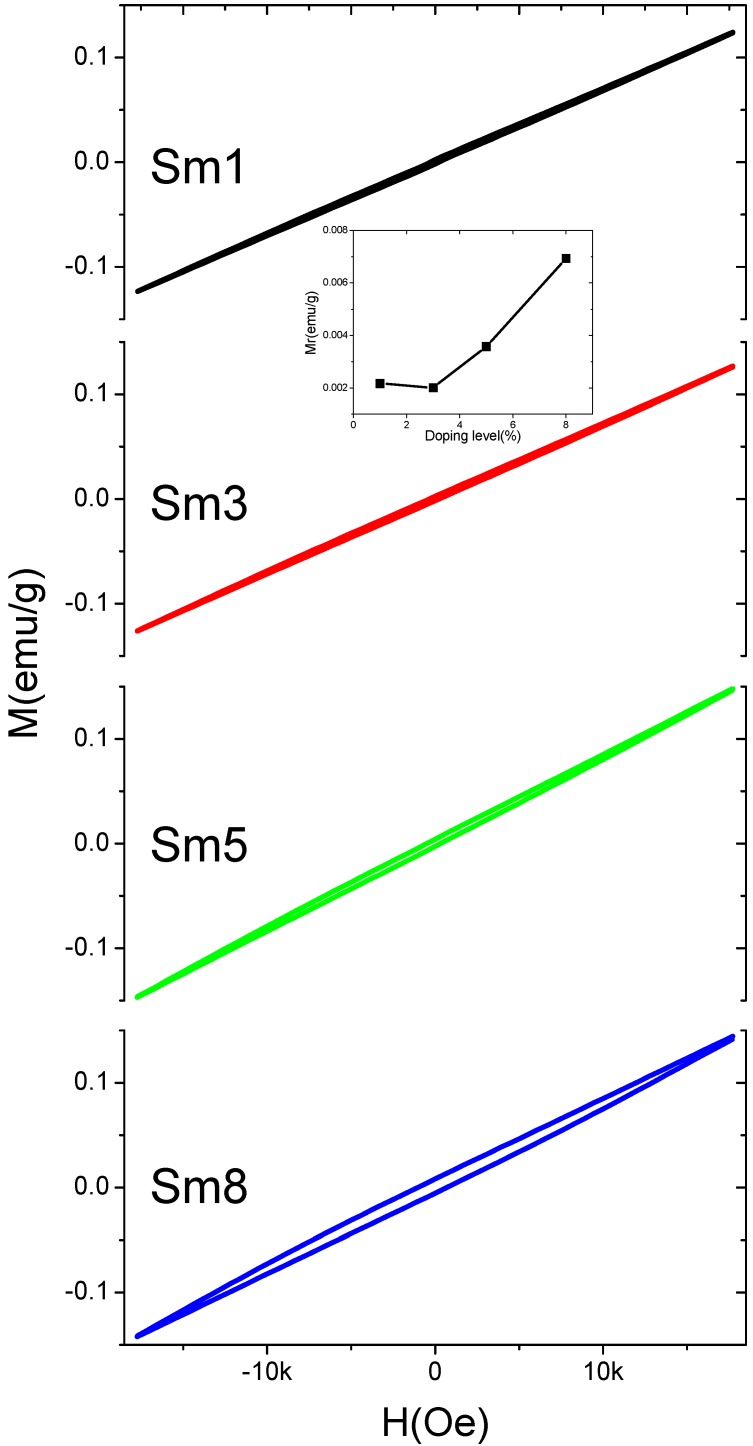
The magnetic M–H loops of the 825 °C-sintered samples. The insets in the figure show the composition dependency of remnant magnetization (M_r_).

Figure 9 shows the magnetization hysteresis (M–H) loops of the 825 °C-sintered samples with a maximum magnetic field (H_m_) of 18 kOe. The remnant magnetization (M_r_) was increased from 0.002 emu/g to 0.007 emu/g, as the Sm doping level increased from 1% to 8%, as shown in the inset of Figure 9. The coercive field (H_c_) of the Sm8 sample is 0.8 kOe. The observation of the remnant magnetization in the doped BFO is believed to be due to the collapse the space modulated antiferromagnetic spin structure [4,10,11,12,13,14,15,16,17,18,19,20,21,22]. Pure BFO shows a magnetic cycloidal spiral structure (G-type antiferromagnetic ordering of Fe^3+^ ions) with a long period of 620 ± 20 Å [37] and the remnant magnetization is zero due to the cancellation of the space-modulated magnetic spins. After introducing a smaller ion at Bi-site, the crystallographic structure is distorted, as shown in the XRD results. The Fe^3+^–O–Fe^3+^ bond will bend due to the structure distortion, and, the antiferromagnetic ordering is then corrupted. It was shown that the remnant magnetization was increased up to 1emu/g in the doped BFO ceramics with a seriously-distorted rhombohedral structure [15]. Besides that, the Sm^3+^ ion is magnetically-active, and it can also be a possible source of the remnant magnetization. The remnant magnetization and polarization is both necessary for ME applications. Bearing the considerably improved ferroelectric and magnetic properties as discussed above, the Sm-doped BFO material shows promise for the ME applications.

## 4. Conclusions

Sm-doped BFO ceramics with low doping levels (1 mol %, 3 mol %, 5 mol %, and 8 mol %) were obtained using a modified conventional solid-state-reaction method. The preparation procedure was modified with a fast heating (30 °C/min) and cooling process (100 °C/min). The sintered samples were annealed in air for 5 h at temperatures from 100 °C to 500 °C. Based upon their dielectric properties, the optimum sintering and annealing temperatures were found to be 825 °C and 300 °C, respectively. The Sm1 sample exhibits the lowest dielectric loss <0.01 at frequencies from 1 kHz to 3 MHz. Leakage-free and saturated ferroelectric P–E loops were observed in all samples. The remnant polarization was maximized in the 835 °C-sintered Sm5 sample (~35 μC/cm^2^). The remnant magnetization appears after Sm doping and the Sm8 shows the largest Mr of 0.007 emu/g with a coercive field of 0.8 kOe. It was found that Sm-dopant can improve the dielectric, ferroelectric, and magnetic properties of BFO ceramics.
